# Comparative Evaluation of Two Microfluidic Sperm Sorting Devices: Laboratory Assessment of Sperm Quality and Retrospective Analysis of Embryological Outcomes Following Intracytoplasmic Sperm Injection

**DOI:** 10.1002/rmb2.70010

**Published:** 2026-01-08

**Authors:** Ryota Tachibana, Hiroki Takeuchi, Miyu Yotsutani, Mikiko Nishioka, Erina Takayama, Eiji Kondo

**Affiliations:** ^1^ Center of Advanced Reproductive Medicine Mie University Hospital Tsu Mie Japan; ^2^ Department of Obstetrics and Gynecology, Graduate School of Medicine Mie University Tsu Mie Japan; ^3^ Obstetrics and Gynecology Mie University Hospital Tsu Mie Japan

**Keywords:** DNA damage, intracytoplasmic sperm injection, microfluidics, sperm capacitation, sperm motility

## Abstract

**Purpose:**

Density gradient centrifugation (DGC) enriches motile sperm but may increase sperm DNA fragmentation (SDF), whereas microfluidic sperm sorting devices, such as ZyMōt and CA0, enable centrifugation‐free selection with lower SDF. Direct comparisons are limited, and their relative clinical efficacy remains unclear. We compared sperm parameters and SDF after DGC, ZyMōt, and CA0 to evaluate sperm selection performance and assessed the clinical utility of ZyMōt and CA0 based on intracytoplasmic sperm injection (ICSI) outcomes.

**Methods:**

Seventeen semen samples were aliquoted into three groups and processed using DGC, ZyMōt, or CA0. Sperm parameters and SDF were evaluated before and after selection. ICSI was performed using ZyMōt or CA0‐selected sperm, inseminating 108 oocytes with ZyMōt and 64 with CA0, followed by the comparative analysis of embryo development.

**Results:**

ZyMōt‐selected sperm exhibited higher motility, average path velocity, and amplitude of lateral head displacement than DGC (*p* < 0.01); CA0‐selected sperm showed higher linearity and wobble. ZyMōt achieved higher rates of fertilization, blastocyst formation, and good‐quality blastocysts, and a lower percentage of poor‐quality cleavage‐stage embryos than CA0 (all *p* < 0.05).

**Conclusions:**

ZyMōt demonstrated superior sperm quality compared with DGC and CA0, and favorable ICSI outcomes further support its potential clinical applicability.

**Trial Registration:**

The study (H2023‐230) was registered in the Japan Registry of Clinical Trials (jRCT) Clinical Trials Registry in Japan (jRCT1040230045)

## 1 Introduction

Male infertility contributes to approximately 50% of infertility cases among couples, with nearly 80% of these cases attributed to spermatogenic dysfunction, including abnormalities in sperm production or maturation, such as oligozoospermia, asthenozoospermia, and teratozoospermia [1]. Advances in assisted reproductive technology (ART) have expanded therapeutic options for difficult‐to‐treat male infertility. In cases with relatively normal semen parameters, in vitro fertilization (IVF) may be sufficient; however, intracytoplasmic sperm injection (ICSI) is the standard approach for severe male infertility characterized by markedly reduced sperm count or motility [2, 3]. In such cases, selecting motile and morphologically normal spermatozoa is crucial for optimizing treatment outcomes [4, 5]. Various sperm selection techniques have been developed to isolate sperm with good motility and morphology, with density gradient centrifugation (DGC) being the most widely used in clinical practice [6, 7].

In DGC, semen is layered on top of multiple density gradients and subjected to centrifugation [8, 9]. Spermatozoa density correlates with maturation status: mature sperm are denser, while immature sperm retain cytoplasmic droplets and have lower density. This density‐based principle allows DGC to effectively separate mature, motile, morphologically normal sperm from immature sperm and leukocytes, thereby enriching a sample with high‐quality sperm [10, 11]. However, the centrifugation process may induce mechanical stress, potentially increasing sperm DNA fragmentation (SDF) [12]. Poor sperm quality and increased SDF are associated with adverse ART outcomes, including embryonic arrest, blastomere fragmentation, and poor embryo quality [13, 14, 15].

To address these limitations, microfluidic sperm‐sorting (MSS), which does not involve centrifugation, has recently gained attention. MSS enables the selection of highly motile sperm by allowing sperm to swim through microchannels that mimic physiological conditions [16, 17]. This approach has been shown to minimize physical damage to sperm and may reduce SDF by avoiding mechanical stress induced by centrifugation [16, 18, 19]. ZyMōt is a commercially available MSS device that improves sperm quality and ART outcomes [20]. Another sperm sorting device, LensHooke CA0, also enhances sperm quality [21]. While both are microfluidic‐based devices, structural and functional differences may influence performance. However, direct comparative studies between ZyMōt and CA0 are limited [21], and their clinical efficacy remains unclear.

This study aimed to compare three sperm selection techniques—DGC, ZyMōt, and CA0—for laboratory evaluation of sperm quality to identify the most effective method for isolating high‐quality sperm in clinical settings. In addition, a separate analysis evaluated the clinical utility of ZyMōt and CA0 by comparing embryological outcomes following ICSI using sperm prepared in these two devices. The results will help elucidate the characteristics of each sperm sorting method and can optimize sperm selection for ART.

## 2 Materials and Methods

### 2.1 Study Design and Ethical Approval

Sperm quality was evaluated using DGC, ZyMōt, and CA0, and ICSI outcomes were compared between ZyMōt and CA0. A total of 54 couples participated in the study, with 17 included in the sperm quality analysis and 36 in the ICSI outcome analysis. For sperm quality assessment, semen samples were collected from patients undergoing fertility treatment at our institution between March 2023 and March 2024. All participants provided written informed consent, as approved by the Mie University Ethics Committee, to ensure adherence to ethical standards (approval number: H2023‐230). For ICSI outcome analysis, clinical data were retrospectively collected from patients treated between April 2024 and June 2025 under an opt‐out consent procedure approved by a separate ethics protocol (approval number: H2024‐083).

### 2.2 Measurement of the Pore Size and Membrane Thickness of MSS Devices

Pore diameter, pore opening area, membrane thickness, and proportion of abnormal pores in the ZyMōt and CA0 MSS devices were measured using a BX43 microscope with a DP74 digital camera and cellSens imaging software version 2.3 (Evident, Tokyo, Japan). Pore diameter and opening area were measured at 400× magnification in randomly selected fields. Fifty pores were manually measured for each device. Membrane thickness was measured by imaging the cross‐section of the porous membrane at 400× magnification. Abnormal pores were defined as structures formed by the fusion of multiple adjacent pores, and their frequency was calculated as the proportion of such pores among 200 measured pores.

### 2.3 Sperm Analysis

Semen samples were obtained from the healthy male partners of 17 female patients with infertility who visited our clinic. After liquefaction for at least 30 min, semen volume was measured, and sperm parameters were analyzed using a computer‐assisted sperm analysis (CASA) system (LensHooke X1 PRO; Bonraybio, Taichung, Taiwan). Although CASA is not explicitly recommended in the WHO laboratory manual, it makes objective and reproducible assessments of sperm motility and concentration. The reliability and validity of the LensHooke CASA system have been demonstrated in a previous study [22]. Sperm in each sample was processed using DGC, ZyMōt, and CA0. The following sperm parameters were evaluated using CASA: semen concentration, sperm count, motility rate, progressive motility rate, number of motile sperm, number of progressively motile sperm, average path velocity (VAP), straight‐line velocity (VSL), curvilinear velocity (VCL), percentage of rapidly motile sperm (VAP ≥ 25 μm/s), percentage of slowly motile sperm (VAP < 25 μm/s), linearity (LIN), straightness (STR), wobble (WOB), amplitude of lateral head displacement (ALH), beat cross frequency, and normal morphology rate. Additionally, sperm head length, head width, head perimeter, head area, and tail length were measured. An aliquot of each sample was reserved for SDF analysis and pretreated for terminal deoxynucleotidyl transferase dUTP nick‐end labeling (TUNEL) staining.

### 2.4 Sperm Separation by DGC, ZyMōt, and CA0

Semen samples were collected via masturbation and allowed to liquefy at room temperature for approximately 30 min. All samples were used for sperm preparation within 2 h after ejaculation to minimize time‐dependent changes in sperm quality. Semen analysis was performed immediately after liquefaction. Only samples with total sperm concentration > 2.0 × 10^6^ cells/mL and motility > 30% were included, and each sample was divided into three aliquots for processing using DGC, ZyMōt, and CA0.

DGC was performed using a two‐layer gradient consisting of 40% and 80% density phases. PureCeption 80% Lower Phase Gradient (CooperSurgical, CT, USA) was used as the lower layer. The 40% upper layer was prepared by diluting the 80% gradient with multipurpose handling medium (MHM; FUJIFILM Irvine Scientific, CA, USA). Samples were centrifuged at 400× *g* for 15 min. The supernatant was discarded, and the sperm pellet was resuspended in MHM supplemented with 10% (v/v) dextran serum supplement (Irvine Scientific, CA, USA) and centrifuged at 400× *g* for 10 min. The supernatant was again discarded.

Sperm selection using ZyMōt (CooperSurgical, CT, USA) was performed according to the manufacturer's instructions. Briefly, 0.85 mL of semen was loaded into the device's inlet port using a 1 mL syringe (Terumo, Tokyo, Japan). Subsequently, 0.75 mL of G‐IVF Plus medium (Vitrolife, Göteborg, Sweden) was aspirated using a 1 mL syringe and added to the outlet port. The device was incubated in a humidified incubator with 5% O_2_ and 5% CO_2_ at 37°C for 30 min. Then, 0.5 mL of G‐IVF medium overlying the micro‐porous membrane was collected using a syringe.

The CA0 device (Bonraybio, Taichung, Taiwan) contains a lower chamber, an upper chamber, and a cover. The upper chamber contains a polycarbonate membrane filter and a collection port for sperm retrieval. First, 1.0 mL of semen was placed into the lower chamber, which was then connected to the upper chamber. Subsequently, 0.9 mL of G‐IVF Plus medium was added to the upper chamber. The device was covered and incubated at 37°C for 30 min. Then, 0.5 mL of the sperm suspension was retrieved from the collection port of the upper chamber. Sperm parameters were evaluated for all three methods using the CASA system.

### 2.5 SDF Analysis Using the TUNEL Assay

SDF was assessed using the TUNEL assay. The assay was performed using flow cytometry according to the instructions provided in the BD Pharmingen APO‐DIRECT Kit (BD Biosciences, CA, USA). The procedure was conducted as described in our previous study [23]. Sperm samples from each method were adjusted to a concentration of 1.0 × 10^6^ cells and fixed in 1 mL of 4% (w/v) paraformaldehyde at 4°C for 30 min. The samples were centrifuged for 5 min, and the supernatant was discarded. Pellets were washed twice with phosphate‐buffered saline and suspended in 1 mL of ice‐cold 70% ethanol. Samples were stored at −20°C for at least 30 min for permeabilization. After centrifugation, ethanol was removed, and the cells were washed twice with wash buffer. A staining solution was added to each sample according to the manufacturer's protocol, followed by incubation at 37°C for 60 min in the dark. Raw sperm (RAW) stained with the staining solution without terminal deoxynucleotidyl transferase served as a negative control. Samples were washed twice with 1.0 mL of rinse buffer and resuspended in 0.3 mL of propidium/ribonuclease solution. Flow cytometry was performed using a MACSQuant Analyzer 16 with MACSQuantify software (Miltenyi Biotec, North Rhine‐Westphalia, Germany). Negative controls were used in all analyses. A minimum of 5000 events per sample were measured using forward and side scatter readings to exclude contaminating cells, and a scatter plot was generated by gating only sperm.

### 2.6 Ovarian Stimulation and Oocyte Retrieval

Ovarian stimulation methods included both natural and stimulated cycles, and drug use was optimized for each patient. In the stimulated cycle, progestin‐primed ovarian stimulation, short, or antagonist protocols were used. For oocyte maturation, human chorionic gonadotropin or antagonist drugs were administered approximately 36 h before oocyte retrieval. Oocytes were retrieved using single‐lumen needles (18G or 19G; Kitasato, Tokyo, Japan) connected to a vacuum pump (Cook Medical, IN, USA) and then transferred to Falcon 14 mL round‐bottom polystyrene test tubes (Corning, NY, USA). Cumulus‐oocyte complexes (COCs) were identified under a stereomicroscope (SMZ18, Nikon, Tokyo, Japan) and precultured in G‐IVF Plus medium for 1–3 h. COCs were removed using Cumulus Remover (Kitazato, Tokyo, Japan) and diluted 1:1 with MHM, followed by assessment of maturation.

### 2.7 ICSI and Oocyte Culture

ICSI outcomes were retrospectively analyzed in two groups of patients who underwent treatment at our institution. Sperm for ICSI were prepared using either ZyMōt or CA0, depending on the period when each method was available in clinical practice; therefore, allocation was non‐random. Semen samples were collected from healthy male partners of female patients undergoing infertility treatment at our institution. The ZyMōt group included 108 mature oocytes retrieved from 22 patients, and the CA0 group included 64 mature oocytes from 14 patients. None of our patients underwent ICSI using both methods. To minimize the influence of the number of retrieved oocytes, all embryological outcomes were analyzed on a per‐oocyte basis.

ICSI was performed using commercially available injection pipettes (Sunlight Medical, FL, USA) and holding pipettes (Kitazato, Shizuoka, Japan). After ICSI, oocytes were cultured for 16–18 h in one of the following media: Continuous Single Culture–NX complete medium (FUJIFILM Irvine Scientific, CA, USA), HiGROW OVIT PLUS (Fuso Industries, Osaka, Japan), or SAGE 1‐Step (CooperSurgical, CT, USA), after which fertilization was assessed by counting the number of pronuclei inside each oocyte. Oocytes were classified as normally fertilized when two pronuclei (2PN) and two polar bodies were present. Abnormal fertilization was defined as the presence of one or more than three pronuclei at the time of fertilization assessment. After pronuclear evaluation, embryos were continuously cultured in the same medium in a humidified incubator at 37°C, 6% CO_2_, and 5% O_2_ until day 6 post‐fertilization. On day 3, embryos were evaluated using the Veeck classification based on the number of blastomeres, their symmetry, and the degree of fragmentation.

Embryos were categorized into three groups using standard day 3 morphological criteria. High‐quality embryos were defined as those with eight cells, minimal fragmentation (< 10%), and uniform blastomere size. Good‐quality embryos were defined as those with more than eight cells and 10%–25% fragmentation, regardless of blastomere symmetry. Embryos that did not meet these criteria were classified as poor‐quality [24]. On days 5 and 6, embryos were evaluated according to the Gardner classification based on blastocoel expansion, hatching status, and the quality of the inner cell mass and trophectoderm. Embryos graded BL3AA‐BB or higher were considered good‐quality blastocysts and were cryopreserved via vitrification. RAW were analyzed using the CASA system.

Sperm suspensions were analyzed for sperm parameters using a Makler chamber (Sefi Medical Instruments, Haifa, Israel). Sperm concentrations and motility rates were calculated according to the methods described by Bjorndahl et al. [25]. To evaluate at least 200 spermatozoa, all cells within 100 counting grids were counted per analysis. Embryo development outcomes were evaluated based on the number of normally fertilized oocytes (2PN). The percentage of cleavage‐stage embryos was defined as the number of cleavage‐stage embryos divided by the number of 2PN embryos. The percentage of good‐quality cleavage‐stage embryos was defined as the number of good‐quality cleavage‐stage embryos divided by the number of 2PN embryos. The blastocyst formation rate was defined as the number of blastocysts divided by the number of 2PN embryos. The percentage of good‐quality blastocysts was defined as the number of good‐quality blastocysts divided by the number of 2PN embryos.

### 2.8 Statistical Analysis

Statistical analyses were performed using GraphPad Prism version 9 (GraphPad Software, La Jolla, CA, USA). Sperm parameters and SDF rates were compared among the groups using Tukey's multiple comparison test. Embryological outcomes were compared using Fisher's exact test. All data are presented as mean ± standard error of the mean.

## 3 Results

### 3.1 Comparison of Porous Membrane Structure

**FIGURE 1:**
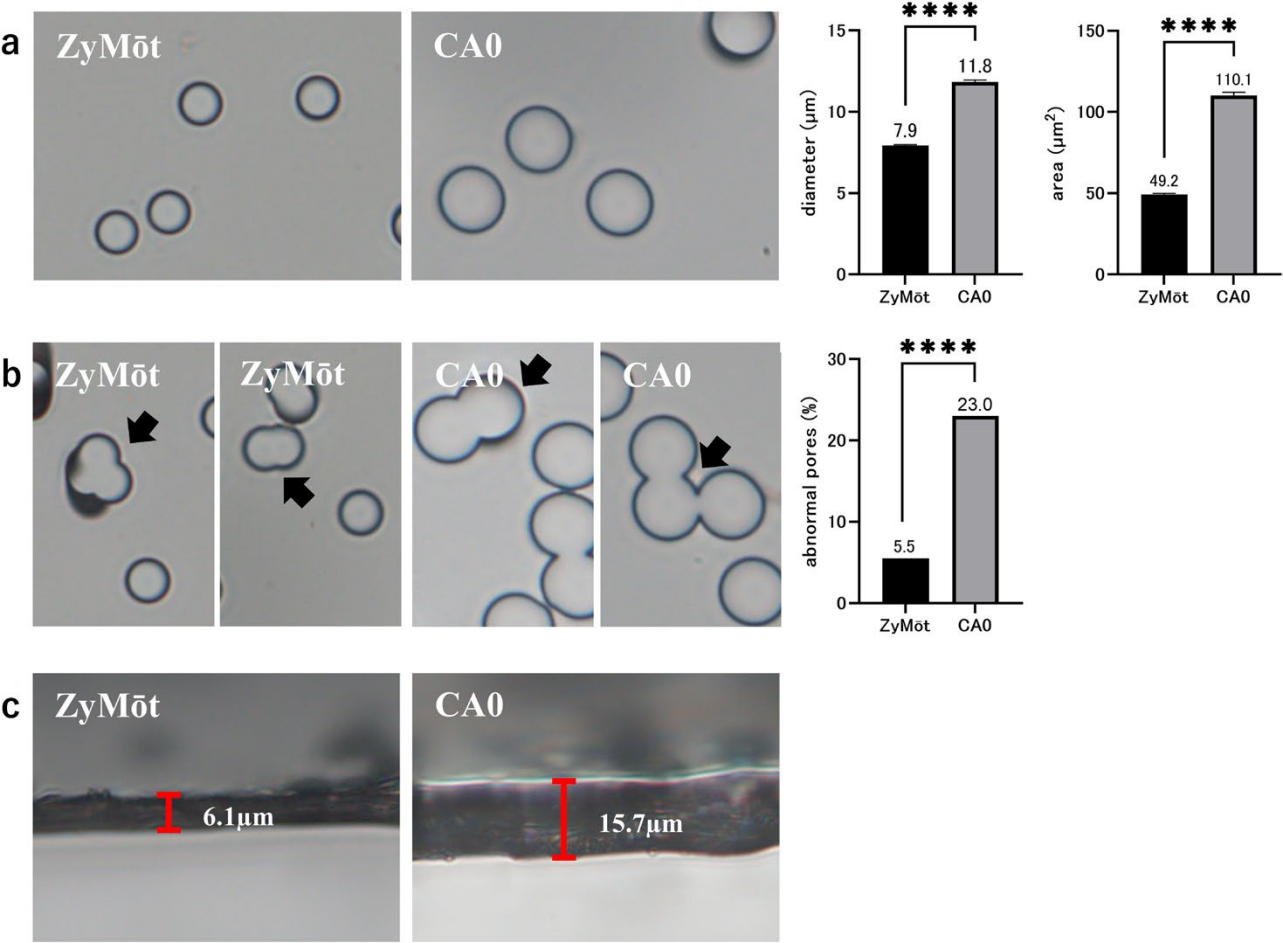
Comparison of the porous membrane structures of ZyMōt and CA0. (a) Representative images of the porous membranes of ZyMōt and CA0 devices. The bar graphs show the comparisons of pore diameter and pore area between the two devices. Pore diameter and opening area were manually measured (50 pores per device). (b) Representative images showing abnormal pores in each membrane. The bar graphs show the proportion of abnormal pores. Abnormal pores were defined as structures formed by the fusion of multiple adjacent pores, and their frequency was calculated as the proportion of such pores among 200 measured pores. (c) Cross‐sectional images of the porous membranes of each device. ZyMōt: ZyMōt sperm separation device; CA0: LensHooke sperm separation device CA0. All images were acquired at 400× magnification. Statistical analysis was performed using an unpaired t‐test for pore diameter and pore area, and Fisher's exact test for the proportion of abnormal pores ****p < 0.0001.

To evaluate the structural characteristics of each MSS device, we measured the pore diameter, pore area, and membrane thickness. The results showed that the average pore diameter was 7.9 ± 0.1 and 11.8 ± 0.1 μm for ZyMōt and CA0, respectively; the pore areas were 49.2 ± 0.1 and 110.1 ± 0.3 μm^2^ for ZyMōt and CA0, respectively (Figure 1a). Both devices exhibited atypical pores formed by the fusion of multiple adjacent pores (Figure 1b). The analysis of 200 pores randomly selected from each device showed that the percentage of abnormal pores was 5.5% and 23.0% in ZyMōt and CA0, respectively (Figure 1b). The membrane thickness of ZyMōt and CA0 was 6.1 and 15.7 μm, respectively (Figure 1c).

### 3.2 Comparison of Sperm Parameters

Sperm quality was assessed by comparing sperm parameters obtained using different processing techniques. Semen characteristics are summarized in Table 1, and the baseline demographic characteristics of the cohort are shown in Table S1. None of the patients had conditions that could affect sperm quality, such as testicular varicocele. SDF rates were assessed using the TUNEL assay (Table 1). The baseline SDF rate in RAW was 18.5% ± 2.7% and significantly decreased to 10.5% ± 2.0% after DGC. MSS‐based selection significantly reduced SDF rates to 3.5% ± 0.7% and 4.1% ± 0.6% with ZyMōt and CA0, respectively. The total sperm count and total motile sperm count were 21.8 ± 4.9 × 10^6^ and 19.6 ± 4.6 × 10^6^ with DGC, 7.5 ± 1.5 × 10^6^ and 7.3 ± 1.4 × 10^6^ with ZyMōt, and 6.5 ± 1.2 × 10^6^ and 6.2 ± 1.2 × 10^6^ with CA0, respectively, with the values of both parameters being significantly higher in the DGC compared to the other methods (Table 1). The sperm recovery rate was significantly higher in the DGC (8.8% ± 1.3%) and ZyMōt (7.6% ± 1.0%) groups than in the CA0 group (5.5% ± 0.8%). The motile sperm recovery rate was significantly higher using ZyMōt (9.6% ± 1.2%) than using CA0 (6.8% ± 0.9%).

**TABLE 1:** Comparison of sperm motility, kinematic parameters, and DNA fragmentation before and after sperm selection using DGC, ZyMōt, and CA0.

Parameters (mean ± SEM)	RAW	DGC	ZyMōt	CA0
Semen volume (mL)	4.4 ± 0.4	—	—	—
Used semen volume (mL)	—	2.1 ± 0.3	0.85	1.0
Recovered semen volume (mL)	—	0.4 ± 0.1	0.5	0.5
Sperm concentration (10^6^/mL)	141.5 ± 19.5^a^	64.3 ± 10.1^b^	15.0 ± 2.9^c^	14.7 ± 2.3^c^
Motile sperm concentration (10^6^/mL)	96.6 ± 14.2^a^	57.8 ± 9.7^a^	14.6 ± 2.8^b^	14.0 ± 2.3^b^
Number of sperm recovered (10^6^ cells)	—	21.8 ± 4.9^a^	7.5 ± 1.5^b^	6.5 ± 1.2^b^
Number of motile sperm recovered (10^6^ cells)	—	19.6 ± 4.6^a^	7.3 ± 1.4^b^	6.2 ± 1.2^b^
Sperm recovery rate (%)	—	8.8 ± 1.3^a^	7.6 ± 1.0^a,b^	5.5 ± 0.8^b^
Motile sperm recovery rate (%)	—	11.1 ± 1.8^a,b^	9.6 ± 1.2^a^	6.8 ± 0.9^b^
SDF value (%)	18.5 ± 2.7^a^	10.5 ± 2.0^b^	3.5 ± 0.7^c^	4.1 ± 0.6^c^

*Note:* Number of sperm recovered = (sperm concentration × recovered semen volume). Number of motile sperm recoverd = (motile sperm concentration × recoverd semen volume). Sperm recovery rate = (number of sperm recovered/number of sperm in RAW used for sperm sorting). Motile sperm recovery rate = (number of motile sperm recovered/number of motile sperm in RAW used for sperm selecting). Values are presented as mean ± standard error of measurement (SEM). Different superscript letters indicate statistically significant differences between groups (*p* < 0.05), as determined by one‐way ANOVA followed by Tukey's post hoc test.

Abbreviation: SDF, sperm DNA fragmentation.

Sperm motility parameters are listed in Table 2. Motility, progressive motility, rapid progressive motility, VAP, VSL, and VCL were significantly higher using ZyMōt than using DGC. LIN was significantly higher in CA0 than in DGC and ZyMōt, and STR was significantly higher in ZyMōt and CA0 than in DGC. WOB was significantly higher in CA0 than in ZyMōt, and ALH was significantly higher in ZyMōt than in DGC and CA0.

**TABLE 2:** Comparison of sperm motility and kinematic parameters among different sperm selection methods.

Parameters (mean ± SEM)	RAW	DGC	ZyMōt	CA0
Total motility (%)	76.2 ± 4.6^c^	88.4 ± 3.1^bc^	98.1 ± 0.3^a^	93.9 ± 1.8^ab^
Progressive motility (%)	60.2 ± 5.2^d^	82.3 ± 4.1^bc^	98.1 ± 0.3^a^	92.8 ± 1.8^ab^
Non progressive motility (%)	15.9 ± 1.9^d^	6.1 ± 1.3^c^	0.1 ± 0.1^a^	1.1 ± 0.6^ab^
Immotility sperm (%)	23.8 ± 4.6^c^	11.6 ± 3.1^bc^	1.9 ± 0.3^a^	6.1 ± 1.8^ab^
Rapid progressive motility (%)	28.6 ± 3.3^d^	54.2 ± 4.5^bc^	78.1 ± 2.4^a^	72.7 ± 3.8^ab^
Slow progressive motility (%)	31.6 ± 2.6^b^	28.1 ± 2.5^ab^	19.9 ± 2.5^ab^	20.1 ± 3.3^a^
VAP (μm/s)	19.5 ± 1.2^c^	31.6 ± 2.3^b^	38.2 ± 1.7^a^	37.0 ± 1.8^ab^
VSL (μm/s)	15.5 ± 1.1^c^	28.0 ± 2.2^b^	34.2 ± 1.6^a^	33.8 ± 1.8^ab^
VCL (μm/s)	28.0 ± 1.7^c^	40.5 ± 2.5^b^	52.9 ± 2.2^a^	47.5 ± 2.0^ab^
LIN (%)	46.6 ± 2.3^c^	62.5 ± 2.3^b^	67.6 ± 3.0^b^	72.2 ± 2.6^a^
STR (%)	66.2 ± 2.1^c^	80.8 ± 1.6^b^	88.3 ± 1.2^a^	89.8 ± 1.2^a^
WOB (%)	63.8 ± 1.7^d^	73.4 ± 1.7^ab^	74.4 ± 2.7^b^	78.7 ± 2.3^a^
ALH (μm)	2.1 ± 0.1^b^	2.4 ± 0.1^b^	3.0 ± 0.2^a^	2.5 ± 0.2^b^
BCF (Hz)	5.7 ± 0.1^b^	6.2 ± 0.2^a^	6.9 ± 0.2^a^	6.5 ± 0.2^a^

*Note:* Values are presented as mean ± standard error of measurement (SEM). Different superscript letters indicate statistically significant differences between groups (*p* < 0.05), as determined by one‐way ANOVA followed by Tukey's post hoc test.

Abbreviations: ALH, Amplitude of Lateral Head displacement; BCF, Beat Cross Frequency; LIN, Linearity; STR, Straightness; VAP, Velocity Average Path; VCL, Curvilinear Velocity; VSL, Velocity Straight Line; WOB, Wobble.

Morphological parameters are presented in Table 3. No significant differences were observed in sperm head and tail measurements among the groups; however, sperm processed with ZyMōt tended to exhibit slightly larger head length and head area. The rate of abnormal head length was significantly higher in sperm processed with ZyMōt than in RAW. The rate of abnormal tail length was significantly lower in all sperm samples compared to RAW.

**TABLE 3:** Comparison of sperm morphology among different sperm selection methods.

Parameters (mean ± SEM)	RAW	DGC	ZyMōt	CA0
Sperm head length (μm)	5.1 ± 0.1	5.2 ± 0.1	5.3 ± 0.1	5.2 ± 0.1
Sperm head width (μm)	3.3 ± 0.1	3.4 ± 0.1	3.4 ± 0.1	3.3 ± 0.1
Sperm head perimeter (μm)	12.7 ± 0.1	13.1 ± 0.1	13.3 ± 0.2	13.0 ± 0.1
Sperm head area (m^2^)	12.8 ± 0.2	13.6 ± 0.2	13.8 ± 0.3	13.5 ± 0.3
Sperm tail length (μm)	16.6 ± 0.9^b^	26.2 ± 1.9^a^	29.1 ± 2.2^a^	31.3 ± 1.3^a^
Normal morphology (%)	4.8 ± 0.3	3.4 ± 0.5	2.7 ± 0.6	4.2 ± 0.6
Head Length abnormal (%)	46.9 ± 2.2^b^	53.1 ± 2.2^ab^	64.1 ± 4.2^a^	54.7 ± 3.3^ab^
Head Width abnormal (%)	48.2 ± 2.0	58.5 ± 4.3	58.4 ± 5.5	50.4 ± 4.5
Head Perimeter abnormal (%)	35.8 ± 2.5	41.7 ± 2.4	47.7 ± 4.8	42.1 ± 4.0
Head Area abnormal (%)	36.4 ± 2.1	48.9 ± 4.0	56.1 ± 5.7	49.4 ± 4.9
Tail Length abnormal (%)	24.1 ± 0.4^a^	17.9 ± 1.0^b^	16.5 ± 1.3^b^	16.5 ± 0.9^b^

*Note:* Values are presented as mean ± standard error of measurement (SEM). Different superscript letters indicate statistically significant differences between groups (*p* < 0.05), as determined by one‐way ANOVA followed by Tukey's post hoc test.

### 3.3 Comparison of Embryological Outcomes

ICSI was performed using sperm selected by ZyMōt or CA0, and embryological outcomes were compared between groups. The baseline characteristics of the 36 couples included in this analysis are summarized in Tables S2 and S3. There were no significant between‐group differences in female age, AMH levels, or ovarian stimulation protocols that could influence the embryological outcomes. ZyMōt was performed on 108 mature oocytes retrieved from 22 patients with infertility, while CA0 was performed on 64 mature oocytes retrieved from 14 patients with infertility (Table 4). There were no significant between‐group differences in abnormal fertilization rate, percentage of cleavage‐stage embryos, percentage of high‐quality cleavage‐stage embryos, and percentage of good‐quality embryos. However, the normal fertilization rate was significantly higher in ZyMōt (85.2%) than in CA0 (71.9%). The percentage of poor‐quality cleavage‐stage embryos was significantly lower in the ZyMōt group (43.5%) than in the CA0 group (65.2%). The blastocyst formation rate was 65.2% and 34.8% in ZyMōt and CA0, respectively, and the good‐quality blastocyst formation rate was 48.9% and 30.4% in ZyMōt and CA0, respectively; both rates were significantly higher in ZyMōt than in CA0.

**TABLE 4:** Embryological outcome following ICSI using sperm selected by different microfluidic sperm sorting methods.

Parameters	ZyMōt	CA0	*p*
	(*n* = 22)	(*n* = 14)	
No. of maturation oocytes	108	64	—
Normal Fertilization (%)	85.2 (92/108)	71.9 (46/64)	< 0.05
Abnormal Fertilization (%)	4.6 (5/108)	6.3 (4/64)	NS
Total cleavage‐stage embryo (%)	95.7 (88/92)	97.8 (45/46)	NS
High‐quality cleavage‐stage embryo (%)	15.2 (14/92)	6.5 (3/46)	NS
Good‐quality cleavage‐stage embryo (%)	37.0 (34/92)	26.1 (12/46)	NS
Poor‐quality cleavage‐stage embryo (%)	43.5 (40/92)	65.2 (30/46)	< 0.05
Total blastocyst formation (%)	65.2 (60/92)	34.8 (16/46)	< 0.05
Good‐quality blastocyst (%)	48.9 (45/92)	30.4 (14/46)	< 0.05

*Note:* Values are presented as mean ± standard error of measurement (SEM) or percentage (%), as appropriate. Fertilization % = (number of normally fertilized oocytes [2PN]/number of inseminated MII oocytes) × 100. Abnormal fertilization % = (number of abnormally fertilized oocytes other than 2PN/number of inseminated MII oocytes) × 100. Total cleavage‐stage embryo % = (number of cleavage‐stage embryos/number of 2PN embryos) × 100. High‐quality cleavage‐stage embryo % = (number of high‐quality cleavage‐stage embryos/number of 2PN embryos) × 100. Good‐quality cleavage‐stage embryo % = (number of good‐quality cleavage‐stage embryos/number of 2PN embryos) × 100. Poor‐quality cleavage‐stage embryos % = (number of poor‐quality cleavage‐stage embryos/number of 2PN embryos) × 100. Total blastocyst formation % = (number of blastocysts/number of 2PN embryos) × 100. Good‐quality blastocyst % = (number of good‐quality blastocysts/number of 2PN embryos) × 100. NS, not significant. Statistical significance indicated by Fisher's exact test (*p* < 0.05).

## 4 Discussion

In this study, we evaluated various parameters of sperm processed using three sperm selection methods—DGC and MSS‐based ZyMōt and CA0. Furthermore, the embryological outcomes of sperm selected using ZyMōt or CA0 were compared.

DGC yielded significantly higher total and motile sperm counts after processing, with recovery rates more than threefold greater than those of ZyMōt and CA0. This outcome is attributed to DGC's ability to process the entire semen volume, which is advantageous for procedures such as intrauterine insemination, where sperm count significantly affects pregnancy rates [26, 27]. In contrast, ICSI requires only a small amount of sperm and hence places greater emphasis on quality over quantity. Therefore, sperm selection in ICSI should involve a comprehensive evaluation of both the quantity and quality of recovered sperm.

Sperm quality indicators include motility, morphology, SDF, and capacitation. Capacitation involves physiological changes such as remodeling of the acrosomal membrane and hyperactivation and can be assessed using sperm kinematic parameters [28]. Specifically, capacitated sperm typically exhibit increased curvilinear VAP, VCL, and ALH, and decreased LIN and WOB [28, 29, 30, 31, 32]. Sperm selected with ZyMōt showed higher VAP, VCL, and ALH, indicating higher capacitation. In contrast, sperm processed with CA0 exhibited higher LIN and WOB, suggesting lower capacitation. One possible explanation for this difference lies in variations in the structural characteristics of porous membranes used in the devices. Compared to ZyMōt, the CA0 membrane has a larger pore diameter, approximately twice the total pore opening area, approximately fourfold the abnormal pore rate, and more than double the membrane thickness. Increased membrane thickness can hinder the migration of hyperactivated sperm due to larger travel distances and higher friction. Furthermore, membranes with larger pore openings or irregular pore structures may increase the risk of seminal plasma contamination during sperm selection, potentially affecting sperm quality. As seminal plasma contains factors that can suppress capacitation, contamination may further impair capacitation [33].

The percentage of sperm with head abnormalities was significantly higher following ZyMōt processing. This may reflect its thin, porous membrane, which facilitates the passage of hyperactivated sperm and sperm with head abnormalities. These findings suggest that ZyMōt effectively selects sperm with high motility but does not completely exclude sperm with morphological abnormalities.

Despite the observed morphological disadvantages, sperm selected using ZyMōt yielded superior embryological outcomes compared to CA0. Specifically, ZyMōt processing yielded a significantly higher normal fertilization rate, a lower proportion of low‐quality cleavage‐stage embryos, a higher blastocyst formation rate, and a higher good‐quality blastocyst formation rate. This difference is likely attributed to variations in seminal plasma contamination, sperm nuclear integrity, and chromatin condensation [34, 35]. Seminal plasma contamination can inhibit sperm capacitation, thereby reducing fertilization rates, and may also cause bacterial contamination, which in turn can impair embryo development [36, 37, 38]. CA0 may favor contamination by seminal plasma, potentially contributing to the observed differences. Furthermore, it is well established that reductions in nuclear integrity and chromatin condensation adversely affect embryonic development rates. Among the sperm selection methods evaluated, ZyMōt yielded the lowest SDF rates and the highest DNA integrity. Additionally, chromatin condensation is positively correlated with sperm motility, suggesting that sperm selected by ZyMōt also possess higher chromatin condensation [35]. While sperm processed using ZyMōt tended to have a larger head area, which is typically associated with chromosomal segregation errors and nuclear maturation defects that could affect fertilization and embryo development [39, 40], these morphological features did not impair fertilization or embryo development rates. This is likely caused by the characteristics of ICSI, where embryologists select morphologically normal sperm under high magnification to minimize the likelihood of using sperm with head abnormalities. Previous studies have shown that mild head abnormalities do not significantly impact fertilization, the percentage of cleavage‐stage embryos, implantation, or live birth outcomes following ICSI [41, 42].

This study has limitations. First, structural details of the sperm selection devices—internal flow dynamics, pore sizes, and membrane materials—were only partially disclosed, limiting the mechanistic interpretation of the observed differences in device performance. Second, although this study assessed sperm parameters and embryological outcomes after sperm selection, it did not evaluate long‐term clinical outcomes such as clinical pregnancy and live birth rates. Future studies should investigate the associations between sperm selection methods and these endpoints to better assess their clinical utility in reproductive medicine. Third, the single‐center design may limit the generalizability of the findings due to potential variations in technical expertise and patient demographics across different facilities. Further validation through multicenter collaborative studies is necessary to confirm these results. Fourth, the semen parameters of our cohort were within nearly normal ranges for both concentration and motility; therefore, it remains unclear whether the observed differences would apply to patients with abnormal semen profiles.

In conclusion, the present study evaluated the performance and clinical outcomes of different sperm selection methods using two complementary analyses. Sperm processed using ZyMōt exhibited higher motility and lower DNA fragmentation than DGC and CA0. Consistently, ICSI cycles using ZyMōt‐selected sperm showed favorable embryological outcomes. Although the cohorts were independent, these findings collectively support the potential clinical advantage of ZyMōt under comparable laboratory conditions.

## Funding

This study was supported by JSPS KAKENHI (Grant Number 24 K19724) and Naka Medical.

## Ethics Statement

All procedures followed were in accordance with the ethical standards of the responsible committee on human experimentation (institutional and national) and with the Declaration of Helsinki of 1964 and its subsequent amendments. The sperm quality study was approved by the Clinical Research Ethics Committee of Mie University Hospital (Approval No. H2023‐230) and complied with the guidelines of the Ethics Committee of the Japan Society of Obstetrics and Gynecology (No. 127). The retrospective analysis of embryological outcomes following ICSI was separately approved by the same committee (Approval H2024‐083) and was conducted using an opt‐out consent procedure.

## Consent

Informed consent was obtained from all patients for being included in the study.

## Conflicts of Interest

H. Takeuchi has received a joint research grant from Naka Medical. The other authors declare no conflicts of interest associated with this manuscript.

## Supporting information


**Table S1:** Patient characteristics used for analyzing sperm parameters.
**Table S2:** Male patient profiles in the study of intracytoplasmic sperm injection outcomes and embryo development.
**Table S3:** Female patient profiles in the study of intracytoplasmic sperm injection outcomes and embryo development.

## Data Availability

The data that support the findings of this study are available from the corresponding author, Hiroki Takeuchi, upon reasonable request.
